# Clinical significance and gene prediction of a novel classification system based on tacrolimus concentration-to-dose ratio in the early post-liver transplant period

**DOI:** 10.3389/fphar.2025.1614753

**Published:** 2025-07-21

**Authors:** Junwei Fan, Peihao Wen, Liyun Yuan, Yan Xia, Shijie Hu, Xiaoqing Zhang, Zhihai Peng

**Affiliations:** ^1^ Department of General Surgery, Shidong Hospital Affiliated to University of Shanghai for Science and Technology, Shanghai, China; ^2^ Department of General Surgery, Shanghai General Hospital Affiliated to Shanghai Jiao Tong University, Shanghai, China; ^3^ Department of Hepatobiliary and Pancreatic Surgery, The First Affiliated Hospital of Zhengzhou University, Zhengzhou, China; ^4^ CAS Key Laboratory of Computational Biology, Bio-Med Big Data Center, Shanghai Institute of Nutrition and Health, University of Chinese Academy of Sciences, Chinese Academy of Sciences, Shanghai, China; ^5^ Department of Pharmacy, The International Peace Maternity and Child Health Hospital, School of Medicine, Shanghai Jiao Tong University, Shanghai, China; ^6^ Department of General Surgery, Xiang’an Hospital of Xiamen University, School of Medicine, Xiamen University, Xiamen, China; ^7^ Organ transplantation Institute of Xiamen University, Fujian Provincial Key Laboratory of Organ and Tissue Regeneration, School of Medicine, Xiamen University, Xiamen, China

**Keywords:** liver transplantation, tacrolimus, gene polymorphism, drug metabolism, personalized medicine

## Abstract

**Background and Aims:**

Classification system of tacrolimus elimination and its clinical significance has not been well described in liver transplantation. This study aimed to present a novel tacrolimus clearance clinical-FIS (Fast-Intermediate-Slow) classification and its gene prediction system.

**Methods:**

Patients from 3 transplant centers were enrolled in this study. All recipients and their corresponding donor livers from center 1 were genotyped using an Affymetrix DMET Plus microarray, and association analysis was performed using trough blood concentration/weight-adjusted-dose ratios (CDR, (ng/mL)/(mg/kg)). The candidate-associated loci were then sequenced in center 2 and center 3 patients for verification.

**Results:**

A clinical classification based on tacrolimus CDR can effectively divide liver transplantation patients into fast elimination (FE), intermediate elimination (IE), and slow elimination (SE) groups, which we called the clinical-FIS classification. Trough blood concentrations in the clinical-SE group during the early postoperative period were higher than those in the clinical-FE and clinical-IE groups, which could lead to delayed recovery of liver (P = 0.0373) and kidney function (P = 0.0135) and a higher infection rate (P = 0.0086). The prediction accuracy of the current CPIC (Clinical Pharmacogenetics Implementation Consortium)-EIP metabolizer classification based on recipient CYP3A5 rs776746 genotype for clinical-FIS classification was only 35.56%. A newly established genetic-EIP classification including major effect genetic factors (donor and recipient CYP3A5 rs776746) and minor effect genetic factors (recipient SULT1E1 rs3775770 and donor SLC7A8 rs7141505) showed 73.2% overall consistency with the former clinical FIS classification.

**Conclusion:**

Our study presented a novel tacrolimus clearance classification, clinical-FIS, and then proposed a novel prospective genetic-EIP classification as a genotyping basis for precisely predicting the clinical-FIS.

## Highlight

Recent guideline using tacrolimus proposed by CPIC attempts to provide information on the association of CYP3A5 genotypes with patient drug metabolic status but does not conform to the genetic characteristics of liver transplantation population. In term of this, we presented a novel tacrolimus clearance clinical-FIS (Fast-Intermediate-Slow) classification, and then proposed a novel prospective genetic-EIP (Extensive-Intermediate-Poor) classification as a genotyping basis for precisely predicting the clinical-FIS, which could possibly become a new clinical guide for tacrolimus regimen in China, and complement, refine, and elucidate clinical genotypes from the perspective of molecular and genetic biology.

## 1 Introduction

Tacrolimus is a calcineurin inhibitor and the main immunosuppressant drug used after solid organ and hematopoietic stem cell transplantation (Ben Brahim et al., 2025; Charlès et al., 2024; Oku et al., 2024). Adequate immunosuppression is essential for suppressing rejection and increasing the survival rate of transplantation; overimmunosuppression can lead to a series of serious adverse drug reactions, such as infection, diabetes, and renal insufficiency. However, the narrow therapeutic index and large interindividual variabilities of tacrolimus complicate its routine dosage adjustment (Brunet et al., 2019; Al-Kofahi et al., 2021; Pei et al., 2023). Dosages between patients also showed sharp differences of up to more than 20-fold (0.5 mg–10 mg/day). Thus, precise personalized dosage is important for minimizing exposure to calcineurin inhibitors and at the same time achieving low-acute rejection rates (Gabrielli et al., 2025).

Therapeutic drug monitoring (TDM) is the most common strategy for immunosuppressive therapy in daily clinical practice and effectively ameliorates drug efficacy and safety (Pattanaik and Monchaud, 2025; Tanathitiphuwarat et al., 2025). At one time, the dosage of tacrolimus was adjusted continuously guided by monitoring trough blood concentrations in the early period after liver transplantation, and then, the individualized dosage was found. After this period, the clinical value of pharmacogenomics decreased, and the frequency of detection of trough blood concentrations of tacrolimus was reduced. However, this “trial and error” process is prone to give rise to rejection or adverse drug reactions. Thus, predicting the tacrolimus clearance rate and providing a prospective dosage within an accurate reference range to achieve immune balance in a short time is crucial in immunosuppressive therapy.

In recent years, pharmacogenomics research has provided an active strategy that can forecast drug metabolism phenotypes according to genotype (Pirmohamed, 2023; Sadee et al., 2023; Strother et al., 2025). Enzymes in the cytochrome P450 (CYP) 3A family are responsible for the oxidative metabolism of tacrolimus (Mevizou et al., 2024; Wang et al., 2025; So et al., 2025). Recent guidelines for the use of tacrolimus proposed by the Clinical Pharmacogenetics Implementation Consortium (CPIC) are trying to provide information relevant to the interpretation of CYP3A5 genotype and dosing. The recipients’ metabolism phenotypes were accordingly assigned to 3 categories, including Extensive Metabolizer (EM, an individual carrying two functional alleles) with CYP3A5 rs776746 AA, Intermediate Metabolizer (IM, an individual carrying one functional allele and one nonfunctional allele) with CYP3A5 rs776746 AG, and Poor Metabolizer (PM, an individual carrying two nonfunctional alleles) with CYP3A5 rs776746GG. This simple CIPC-EIP classification for tacrolimus is commonly recommended after many organ transplant operations, including kidney, heart, lung, and hematopoietic stem cell transplants, and liver transplants in which the donor and recipient genotypes are identical (Rod et al., 2022). However, almost all clinical liver transplantation is allogeneic, and the genotypes of the recipients and their corresponding donors were different.

Importantly, the current CPIC-EIP classification, which includes recipient CYP3A5 genotypes, has its own limitations in its application to allogeneic liver transplantation for the following reasons: 1) liver and intestine are the two main metabolic organs of tacrolimus (Rod et al., 2022; Mishima et al., 2024). Nevertheless, most recent related studies have mainly focused on renal transplant patients. For liver transplantation patients, the genotypes of the donor liver and recipient intestine are different; the integration of two sets of genomes complicates genetic factors impacting tacrolimus metabolism. 2) Liver function is recovered through the mechanism of liver regeneration 1 or 2 weeks after transplantation and tends to be stable in the third and fourth weeks. Therefore, related studies should be conducted during the first month. However, most of the current research is not conducted during this period. 3) Considerable genetic diversity indeed exists between Chinese individuals and those of other races, which may lead to differential clinical performance. Based on all of these considerations, there is an urgent need to construct a new and comprehensive classification based on genetic differences between different populations in order to guide personalized tacrolimus dosage for the Chinese population.

**FIGURE 1:**
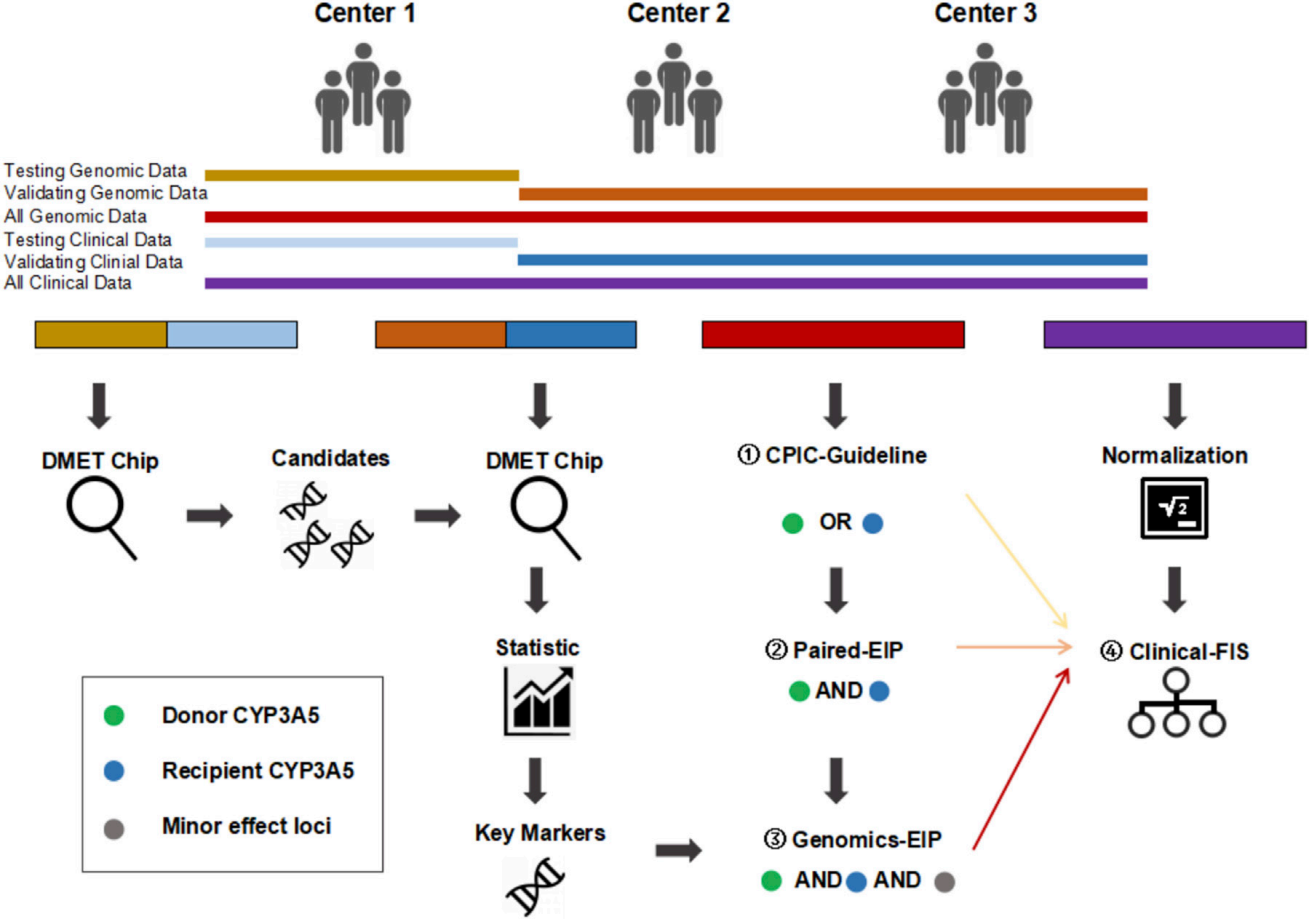
Overall research framework. CPIC-EIP, paired-EIP, and genetic-EIP classification were established base on both genetic and clinical information successively and compared with clinical-FIS classification based only on clinical data. ① CPIC-EIP proposed by Clinical Pharmacogenetics Implementation Consortium includes donor or recipient CYP3A5 rs776746 loci only. ② paired-EIP were validated in our study includes both donor and recipient CYP3A5 rs776746 loci. ③ Genomics-EIP presented in this study includes other minor effect loci besides donor and recipient CYP3A5. ④ Clinical-FIS were firstly proposed in this study and only based on clinical CDR.

In this study, a retrospective clinical-FIS classification was first established based on trough blood concentration/weight-adjusted dose ratios (CDRs, (ng/mL)/(mg/kg)) to divide patients into different subgroups on the basis of important clinical features, including fast elimination (FE), intermediate elimination (IE), and slow elimination (SE) (see Figure 1). Then, a comprehensive dataset including both clinical and genomic data of 284 Chinese liver transplantation patients was collected from 3 independent liver transplant centers (Center 1, Shanghai General Hospital Affiliated to Shanghai Jiao Tong University (n = 114); Center 2, the First Affiliated Hospital of Zhengzhou University (n = 93); Center 3, the First Affiliated Hospital of Medical School of Zhejiang University (n = 77)). In addition to the CYP3A5 genotype, an Affymetrix DMET Plus microarray, a popular DNA microarray platform, was used to investigate broad coverage of pharmacogenomic markers in Center 1 patients, including both donors and recipients. The candidate loci were then validated in Center 2 and Center 3 patient samples. Finally, the new prospective genetic-EIP classification integrated with major and minor effect loci showed high concordance with the retrospective clinical-FIS classification. Based on more comprehensive and systematic genotyping, this study aimed to provide a useful classification to guide tacrolimus dosing in the very early period after liver transplantation, especially for the Chinese population. Additionally, an easy-to-use panel based on this newly established genetic-EIP classification is planned to be designed for further clinical trials.

## 2 Patients and methods

### 2.1 Patients

A total of 284 patients who underwent orthotopic liver transplantation between January 2015 and December 2017 were enrolled. Three independent cohorts were examined: Center 1, Shanghai General Hospital Affiliated to Shanghai Jiao Tong University (n = 114); Center 2, First Affiliated Hospital of Zhengzhou University (n = 93); Center 3, First Affiliated Hospital of Medical School of Zhejiang University (n = 77). The inclusion criteria were (i) adult (≥18 years) patients; (ii) deceased donor primary LT; and (iii) patients who received a tacrolimus-based immunosuppressive regimen. The exclusion criteria were as follows: (i) multiorgan transplant patients; (ii) follow-up time less than 1 month; and (iii) incomplete patient data. Anti-rejection regimens of liver transplantation recipients included tacrolimus, mycophenolate mofetil, baliximab and methylprednisolone. Tacrolimus was administered orally twice daily after the operation. A 1,000 mg dose of mycophenolate mofetil (MMF) was given before the operation, and the postoperative MMF dosage was 750 mg at each administration twice a day. Five hundred milligrams of methylprednisolone were given intravenously before the portal vein was reopened intraoperatively once. 20 mg basiliximab were intravenous injected on day 1 and day 4 post transplantation.

### 2.2 Samples and data collection

Liver samples were collected for genotypic detection. Recipient liver tissues were obtained from diseased liver that had been removed during transplantation, and nontumor liver tissues from at least 5 cm outside the edge of tumors were sampled in patients with hepatic malignant tumors. Donor liver tissues came from zero-time liver biopsy, which is a routine means to assess the quality of donor organs.

The pharmacological parameters of tacrolimus included daily dose and drug trough blood concentration. The blood samples for tacrolimus monitoring were collected before the morning administration. Serial tacrolimus levels in human whole blood were measured daily from postoperative day 1 through day 28 using the Abbott ARCHITECT^®^ chemiluminescent microparticle immunoassay (CMIA) (Abbott Diagnostics, Chicago, IL, United States). The tacrolimus CDR was used as an index of tacrolimus pharmacokinetics and was calculated by dividing the tacrolimus trough blood concentration (ng/mL) by the corresponding weight-adjusted dosage (mg/kg body weight).

Clinical parameters included complete blood cell count and biochemical indicators, such as hemoglobin (Hb), hematocrit (HCT), albumin (Alb), alanine aminotransferase (ALT), aspartate aminotransferase (AST), total bilirubin (TBIL), and direct bilirubin (DBIL), creatinine (Cr), and blood urea nitrogen (BUN) were also recorded in this study.

### 2.3.Genomic DNA isolation and genotyping

Both donor and recipient genomic DNA were extracted from liver tissues (stored at −80°C) using an AllPrep DNA/RNA Mini Kit (Qiagen, Hilden, Germany). The Affymetrix^®^ DMET™ Plus Premier Pack (DMET, Drug Metabolizing Enzymes and Transporters) enables highly multiplexed genotyping of known polymorphisms in absorption, distribution, metabolism, and elimination (ADME)-related genes on a single array. The DMET Plus Panel interrogates markers in 225 genes that have documented functional significance in phase I and phase II drug metabolism enzymes as well as drug transporters. To screen the polymorphic biomarkers associated with tacrolimus elimination, single nucleotide polymorphism data were obtained for the liver donors and 114 recipients from Shanghai General Hospital Affiliated to Shanghai Jiao Tong University using the DMET Plus microarray (Affymetrix, CA, United States). The genotyping procedure was performed according to the DMET Plus Premier Pack protocol.

To verify the SNP screening from the DMET platform, liver donors and recipients in an independent group from the First Affiliated Hospital of Zhengzhou University and First Affiliated Hospital of Medical School of Zhejiang University were genotyped using the Sequenom MassARRAY SNP-genotyping platform (Sequenom, CA, United States). The protocols included PCR amplification, shrimp alkaline phosphatase treatment, single-base extension reaction, resin cleanup, nanodispensing on a SpectroCHIP and data acquisition.

### 2.4. Loci filtering

Loci were retained after filtering in accordance with the following criteria: 1) A P value less than 0.05 continuously in more than 2 weeks; 2) SNPs have a MAF>0.1, because of limited samples; 3) After considering linkage disequilibrium, only one locus of SNPS in LD with R-square >0.5 remained; and 4) Exonic and UTR SNPs were preferred over intronic ones.

A rigorous two-tiered approach to minimize false-positive associations: Training Set Pre-Filtering (n = 114): SNPs were retained based on the Loci filtering criteria; Independent Validation (n = 170): All candidate SNPs from the training phase underwent replication testing in an independent cohort.

### 2.5.Statistical analysis

Genetic Background Data Analysis of populations in the 1000 Genomes Project: A total of 1,136 of the 1931 loci on the DMET array were found in 1000 Genomes Project data (phase 3), and alternative allele frequencies were scanned in 5 independent populations, including African (AFR), Ad Mixed American (AMR), East Asian (EAS), European (EUR), and South Asian (SAS). The allele frequency differences among populations were defined as the highest frequency minus the lowest frequency, i.e., Diff = MAX (MAF)-MIN(MAF).

T-tests were used to assess the statistical significance of differences in CDRs for comparisons of all groups. Correlations between gene polymorphisms and CDRs were studied with univariate and multivariate linear regression analysis. Statistical analysis was performed using R (v4.5.0, https://www.R-project.org/, R Foundation for Statistical Computing, Vienna, Austria). Hardy–Weinberg equilibrium, allele frequency, linkage disequilibrium and haplotype analysis were analyzed using PLINK software. A P value <0.05 was considered indicative of statistical significance.

## 3 Results

### 3.1 Characteristics of tacrolimus administration during the early postoperative period after liver transplantation in the Chinese population

Information from patients who underwent TDM-guided dose adjustments at 28 days postoperatively, including sex, weight and clinical variables such as drug dosage, trough blood concentrations, alanine aminotransferase and total bilirubin, was gathered from three centers and used as phenotypes for further association analysis in pharmacogenomics research (Table 1; Supplementary Figure S1). From the patterns obtained from the above clinical phenotypes, we have seen that the Chinese population has the following clinical characteristics with tacrolimus use: i) the first month trough blood concentration of tacrolimus in the Chinese population varied between 6 ng/mL and 8 ng/mL on the majority of days included in our data. The target therapeutic window within the first 3 months of tacrolimus use was 10–15 ng/mL in the majority of existing literature. Under exposure to the low concentration of tacrolimus, the median alanine aminotransferase level recovered to normal (lower than 40 U/L) on the 10th day postoperatively, and the median total bilirubin was lower than 34 μmol/L (the critical value indicated obvious jaundice) on the eighth postoperative day. ii) The daily dose of tacrolimus gradually increased in the first and second weeks postoperatively and reached the stationary phase (S. phase), in which the daily dose was approximately 0.06 mg/kg. The CDR gradually decreased in the first and second weeks postoperatively and reached a plateau period in which the CDR was approximately 100 (ng/mL)/(mg/kg).

**TABLE 1:** Demographic data for samples from three centers^a^.

Clinical parameters	Center 1 (114)	Center 2 (93)	Center 3 (77)
Age (yr)	47.5 ± 9.01	49.6 ± 9.65	48.8 ± 10.7
Recipient Male/Female (n)	96/18	76/17	61/16
Recipient Weight (kg)	67.7 ± 11.2	65.6 ± 11.1	66.0 ± 11.9
Weight-adjusted Dose (mg/kg)			
Week 1	0.034 (0.039 ± 0.023)	0.038 (0.045 ± 0.030)	0.031 (0.039 ± 0.027)
Week 2	0.048 (0.053 ± 0.031)	0.058 (0.068 ± 0.044)	0.056 (0.058 ± 0.034)
Week 3	0.058 (0.061 ± 0.033)	0.073 (0.076 ± 0.048)	0.057 (0.059 ± 0.039)
Week 4	0.058 (0.061 ± 0.036)	0.069 (0.073 ± 0.045)	0.053 (0.061 ± 0.041)
Month 1	0.048 (0.053 ± 0.033)	0.058 (0.065 ± 0.043)	0.049 (0.055 ± 0.038)
CDR (ng/mL)/(mg/kg))			
Week 1	228 (317 ± 291)	147 (190 ± 136)	122 (143 ± 99)
Week 2	123 (174 ± 173)	82 (106 ± 83)	140 (204 ± 191)
Week 3	109 (166 ± 165)	75 (117 ± 133)	147 (179 ± 127)
Week 4	120 (177 ± 203)	88 (118 ± 144)	111 (153 ± 132)
Month 1	130 (208 ± 222)	91 (134 ± 129)	128 (171 ± 144)
Log (CDR)			
Week 1	5.43 (5.42 ± 0.84)	4.99 (4.99 ± 0.74)	4.80 (4.75 ± 0.67)
Week 2	4.81 (4.88 ± 0.70)	4.41 (4.45 ± 0.63)	4.94 (5.02 ± 0.76)
Week 3	4.69 (4.80 ± 0.73)	4.32 (4.44 ± 0.73)	5.99 (4.96 ± 0.67)
Week 4	4.79 (4.86 ± 0.71)	4.47 (4.51 ± 0.64)	4.71 (4.77 ± 0.70)
Month 1	4.87 (4.99 ± 0.78)	4.51 (4.60 ± 0.72)	4.85 (4.88 ± 0.71)
1st, 3rd quartile	4.43, 5.47	4.09, 4.99	4.38, 5.33
Total Bilirubin (µmol/L)			
Week 1	57 (79.9 ± 69.3)	52.4 (75.2 ± 66.0)	61 (83.5 ± 69.7)
Week 2	27.7 (60.7 ± 84.9)	25.7 (51.4 ± 73.1)	33 (50.5 ± 48.7)
Week 3	20.7 (43.2 ± 66.9)	18.7 (36.0 ± 46.6)	26 (42.5 ± 53.5)
Week 4	21.2 (35.3 ± 38.3)	18.3 (37.8 ± 56.0)	22 (30.6 ± 31.2)
Month 1	31.7 (57.3 ± 71.1)	29.5 (52.5 ± 64.4)	36 (60.5 ± 61.3)

^a^ The numbers presented are median (mean ± standard deviation).

The trough blood concentration of tacrolimus appeared to fluctuate noticeably in the first week after liver transplantation and finally levelled out at 6–8 ng/mL. In the first week postoperatively, CDRs in recipients with the CYP3A5 rs776746 GG genotype were higher than those in recipients with CYP3A5 rs776746 AA/AG, and the CDRs in recipients carrying the donor CYP3A5 rs776746GG genotype were higher than those in recipients carrying the donor CYP3A5 rs776746 AA/AG genotype. The median trough blood concentration of 34.4 (22/64) poor drug metabolizers was over 10 ng/mL at 1 week postoperatively, which could lead to adverse drug reactions. Conversely, the median trough blood concentration of some extensive metabolizers was below 6 ng/mL at 1 week postoperatively, which could lead to rejection. In general, CDRs increased on days 2–3, had a marked decline on days 3–11, and finally stabilized at approximately 100 (ng/mL)/(mg/kg).

### 3.2 A new clinical-FIS classification of tacrolimus metabolism

Looking back at the history of drug use of all the patients, variations in tacrolimus clearance rate existed between individuals. From the clinical performance and dose for immune balance, patients can be divided into three categories according to their tacrolimus clearance, fast elimination (FE) group, intermediate elimination (IE) group, and slow elimination (SE) group. As the patients included in our study came from three different centers, artificial error may be introduced in data collection. Other factors during the whole perioperative period may affect the distribution of final CDRs (see Table 1). Thus, before further analysis and group data integration, CDRs were normalized by logarithmic transformation (Supplementary Figure S2). As the CDRs were continuously recorded during the first month after surgery, four weekly median CDRs were used to reflect the drug elimination rate for four independent weeks. Then, if more than half weekly CDRs were larger than that of the third quartile of 1 month at each independent center, the patient was assigned to the clinical-SE group. Otherwise, if more than half weekly CDRs were smaller than CDRs of the first quartile of 1 month at each independent center, the patient was assigned to the clinical-FE group. The rest of the patients were assigned to the clinical-IE group.

**FIGURE 2:**
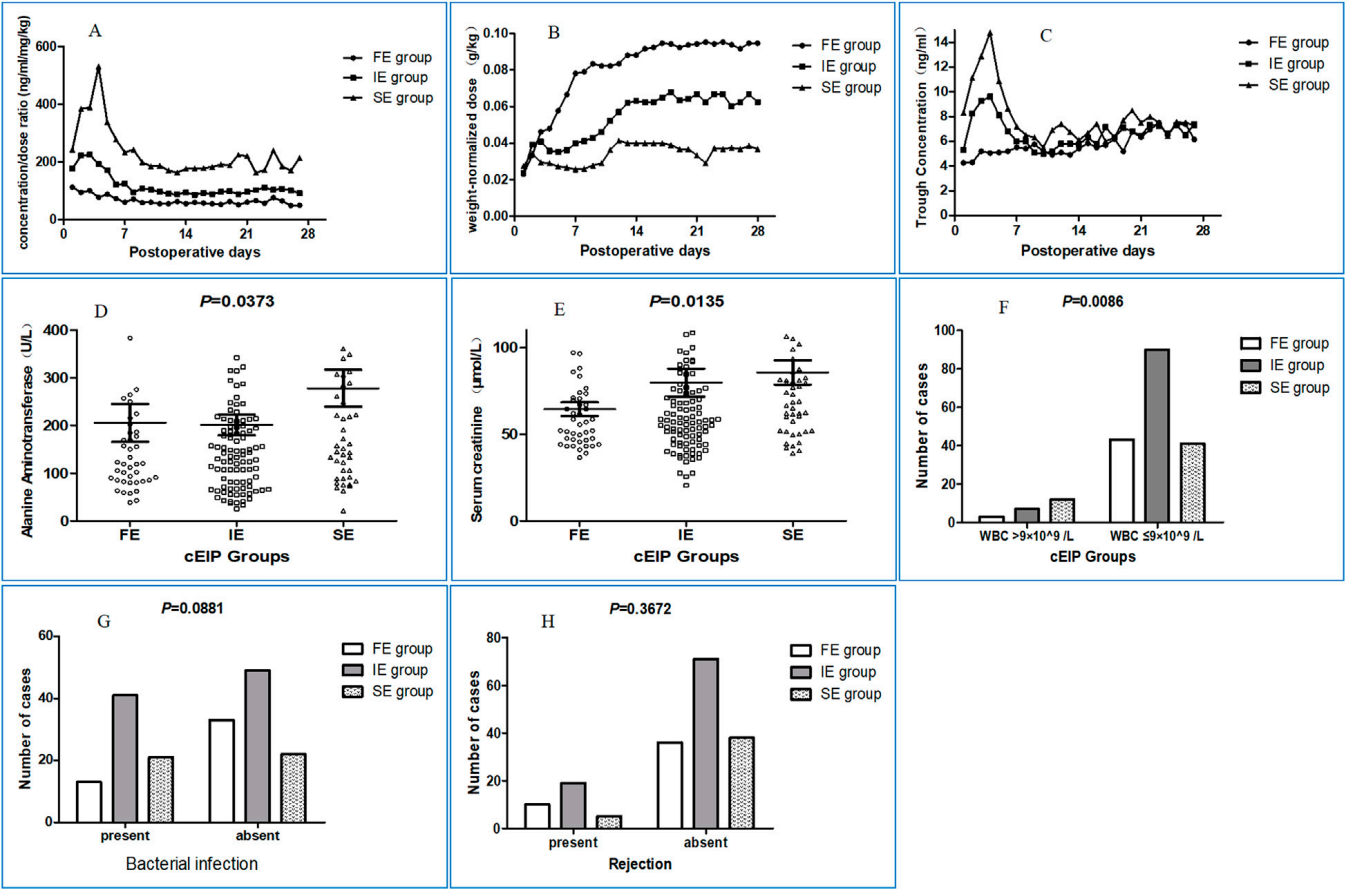
Clinical characteristics of patients in different groups under clinical-FIS classification. **(A)** The changing curves of dose-adjusted trough blood concentrations in FE, IE and SE group at 28 days postoperatively. **(B)** The weight-adjusted dose of each group based on this retrospective clinical-FIS classification can be effectively distinguished. The dose of tacrolimus was low at the outset and was generally increased 1 or 2 weeks after the operation, reaching a plateau. **(C)** The changing curves of concentrations in FE, IE and SE group at 28 days postoperatively. **(D)** The median of alanine aminotransferase in clinical-FE, clinical-IE and clinical-SE group patients in the first postoperative week was 121.7 U/L, 146.4 U/L and 191.1 U/L respectively, with significant differences (P = 0.0373) among the three groups. **(E)** The median of serum creatinine in clinical-FE, clinical-IE and clinical-SE group patients in the first postoperative week was 57.75 μmol/L, 58.5 μmol/L and 74.0 μmol/L respectively, also with significant differences (P = 0.0135) among the three groups. If you prefer, you can place both the actual figures and captions logically through the text near where they are cited rather than at the end of the file (but not both). **(F)** The percentage of recipients carrying higher leukocyte count in clinical-FE (6.52%, 3/46), clinical-IE (7.22%, 7/97) and clinical-SE (22.6%, 12/53) group patients in the first postoperative week than normal, with significant differences among the three groups (P = 0.0086). **(G)** The percentage of recipients with bacterial infection in clinical-FE (28.3%, 13/46), was obviously lower than that in clinical-IE (45.6%, 41/90) and clinical-SE (48.8%, 21/43) group respectively, and there were no significant differences among the three groups (P = 0.0881). **(H)** The percentage of recipients with rejection reaction in clinical-FE, clinical-IE and clinical-SE group patients in the follow-up period was 21.7% (10/46), 21.1% (19/90) and 11.6% (5/43) respectively, and there were no significant differences among the three groups (P = 0.3672).

The weight-adjusted dose of each group based on this retrospective clinical-FIS classification can be effectively distinguished. The dose of tacrolimus was low at the outset and was generally increased 1 or 2 weeks after the operation, reaching a plateau. The stable doses of tacrolimus were 0.101 ± 0.036 mg/kg, 0.068 ± 0.039 mg/kg, and 0.038 ± 0.029 mg/kg in the clinical-FE group, clinical-IE group and clinical-SE group, respectively (Supplementary Figure S3). Moreover, the clinical-SE group had poorer therapeutic effects and more adverse reactions than the clinical-FE and clinical-IE groups (Figure 2). Besides these, we also found that 1) The median of alanine aminotransferase in clinical-FE, clinical-IE and clinical-SE group patients in the first postoperative week was 121.7 U/L, 146.4 U/L and 191.1 U/L respectively, with significant differences (P = 0.0373) among the three groups. 2) The median of serum creatinine in clinical-FE, clinical-IE and clinical-SE group patients in the first postoperative week was 57.75 μmol/L, 58.5 μmol/L and 74.0 μmol/L respectively, also with significant differences (P = 0.0135) among the three groups. 3) The percentage of recipients carrying higher leukocyte count in clinical-FE (6.52%, 3/46), clinical-IE (7.22%, 7/97) and clinical-SE (22.6%, 12/53) group patients in the first postoperative week than normal, with significant differences among the three groups (P = 0.0086). 4) The percentage of recipients with bacterial infection in clinical-FE [28.3% (13/46)] was obviously lower that in clinical-IE [45.6% (41/90)] and clinical-SE [48.8% (21/43)] group in the follow-up period, and respectively, and There is close to statistical significance among the three groups (P = 0.0881). 5) The percentage of recipients with rejection reaction in clinical-FE, clinical-IE and clinical-SE group patients in the follow-up period was 21.7% (10/46), 21.1% (19/90) and 11.6% (5/43) respectively, and there were no significant differences among the three groups (P = 0.3672). This retrospective clinical-FIS classification exhibited good performance in dividing patients into different tacrolimus clearance rate categories, and has become an excellent reference for the following established prospective genetic-EIP classification.

### 3.3 Genotyping of center 1 patients with DMET™ plus microarray

114 donor/recipient pairs in Group 1 were tested using the Affymetrix DMET platform. A total of 1921 SNPs were detected, 752 of which remained with MAF>0. The number of rare variants (MAF<0.01), low frequency variants (0.01<MAF<0.05) and common frequency variants (MAF>0.05) were 89, 126 and 537, respectively. DMET loci were also scanned and found to have obvious variations in distribution in 5 different populations from 1000G (Supplementary Table S1). Loci with high Diff values may explain the genetic differences in clinical medication responses between the Chinese and Caucasian populations, the latter being the main race on which current international guidance is based. CDRs after logarithmic transformation and SNP genotypes were used for association analysis of 114 patients at Center 1, and CYP3A5 rs776746 on both donor and recipient were found to have the most significant differences. The combination of paired genotypes with both donor and recipient CYP3A5 was considered and verified to be more effective in separating patients’ tacrolimus clearance rates than single donor or recipient CYP3A5 (Supplementary Figure S4).

We first combined donor-recipient paired CYP3A5 rs776746 polymorphisms to divide the liver transplantation patients into 4 subgroups: i) donor CYP3A5 rs776746 AA/AG + recipient CYP3A5 rs776746 AA/AG, ii) donor CYP3A5 rs776746 AA/AG + recipient CYP3A5 rs776746GG, iii) donor CYP3A5 rs776746GG + recipient CYP3A5 rs776746 AA/AG, and vi) donor CYP3A5 rs776746GG + recipient CYP3A5 rs776746 GG. Considering that the CDRs of subgroups ii and iii had no significant difference, these two subgroups were incorporated into one; the resulting groups were paired extensive metabolizer (paired-EM, recipient CYP3A5 rs776746 AA/AG and donor CYP3A5 rs776746 AA/AG), paired intermediate metabolizer (paired-IM, recipient CYP3A5 rs776746GG and donor CYP3A5 rs776746 AA/AG, or recipient CYP3A5 rs776746 AA/AG and donor CYP3A5 rs776746GG donor), and paired poor metabolizer (paired-PM, recipient CYP3A5 rs776746GG and donor CYP3A5 rs776746GG).

The stable dose of tacrolimus ranged from 0.080 ± 0.036 mg/kg, 0.068 ± 0.033 mg/kg, and 0.047 ± 0.024 mg/kg in paired-EMs, paired-IMs and paired-PMs, respectively. Compared with the retrospective clinical-FIS classification, this paired-EIP classification based on paired CYP3A5 genotypes did not perform well. Although the paired-EIP classification had a stronger distinguishing ability of tacrolimus dosage than donor CYP3A5 rs776746 or recipient CYP3A5 rs776746, the changes in drug distribution among the three groups were not as significant as expected. As shown in the diagram, some patients with the paired CYP3A5 genotype (donor GG + recipient GG) also had a relatively fast clearance rate, indicating that there may exist certain minor loci that contribute to tacrolimus clearance. In consideration of this factor, association analysis was performed in paired-EMs, paired-IMs, and paired-PMs independently in the same way (Supplementary Material S2), and 31 loci were finally filtered for further validation (see Methods).

### 3.4 Validating the effect of multiple loci on independent patients from centers 2 and 3

31 loci were genotyped using the Sequenom MassARRAY SNP-genotyping platform (Sequenom, CA, United States) with donor and recipient genomic DNA from 170 patients from Centers 2 and 3. Similarly, association analyses were performed on all patients, as well as on the EM, IM, and PM groups. The results for both the test patients from Center 1 (114) and validation patients (170) from Centers 2 and 3 are shown in Table 2 and Supplementary Material S3. A total of 10 loci on 8 genes were ultimately screened out with a clear correlation with tacrolimus clearance. Considering the major effect of rs776746 on CYP3A5 in all patients, a multiple linear regression model was established to define the optimal combination with other loci that also impacted all patients. The results of regression analysis proved that rs7853758 on SLC28A3 and rs914189 on ABCG1 are significant variables and that their joint probabilities with donor and recipient rs776746 on CYP3A5 in the first 4 weeks are 15.88%, 17.92%, 21.76% and 19.2%, which is increased slightly compared with paired rs776746 on CYP3A5 only (Table 3). These findings suggested the above loci have in fact impacted tacrolimus clearance even in strong influence of CYP3A5 in all patients.

**TABLE 2:** T.test results of loci in testing (Center 1) and validating (Center 2 and 3) sample sets.

EIP Group	CHR	SNP	Gene	GT1	GT2	Region	MAF	Testing samples (114)^a^				Validating samples (170)^a^			
								Week1	Week2	Week3	Week4	Week1	Week2	Week3	Week4
All	7	D.rs776746	CYP3A5	AA/AG	GG	splicing	0.28	0.0005	0.0411	0.0044	4.0E-05	0.0056	0.0019	0.0010	0.0105
	7	R.rs776746	CYP3A5	AA/AG	GG	splicing	0.28	0.0021	0.0140	0.0095	0.0198	0.0001	4.3E-05	3.4E-07	4.8E-05
	7	R.rs2242480	CYP3A4	AA/AG	GG	intronic	0.25	0.0162	0.0103	0.0015	0.0027	0.0102	0.0656	0.0017	0.0172
	9	R.rs7853758	SLC28A3	TT/TC	CC	exonic(N)	0.14	0.6449	0.0374	0.0047	0.0017	0.6441	0.0571	0.1847	0.2920
	21	D.rs1541290	ABCG1	GG/GA	AA	intergenic	0.44	0.4685	0.4162	0.0169	0.0826	0.2816	0.0872	0.1036	0.1762
	21	R.rs914189	ABCG1	GG	CC/CG	intronic	0.27	0.9992	0.0034	0.0161	0.0194	0.62285	0.0229	0.0836	0.3934
Paired-EMs	4	R.rs3775770	SULT1E1	AA/AG	GG	intronic	0.22	0.1954	0.0495	0.0959	0.1545	0.6573	0.0825	0.0877	0.1973
Paired-PMs	2	R.rs3748930	CHST10	CC	GG/GC	exonic(S)	0.2707	0.0366	0.1001	0.2067	0.1952	0.1470	0.0624	0.0987	NA
	11	D.rs895729	CHST1	TT/TC	CC	intronic	0.1725	0.1805	0.0069	0.0169	0.0471	0.0778	0.0123	0.1005	0.5548
	14	D.rs7141505	SLC7A8	GG/GT	TT	upstream	0.1594	0.6655	0.3814	0.0425	0.0434	0.6911	0.2224	0.0153	0.0007

^a^ The P-value for the significance of differences in CDR, comparison between GT1 and GT2.

**TABLE 3:** Optimal multiple linear regression model in all patients.

Week	Model with donor and recipient rs776746		Optimal models with minor effect loci	
	SNPs with P-value	Adjusted R^2^	+ SNPs with P-value	Adjusted R^2^
1	D.rs776746 (5.4e-07) + R.rs776746 (1.1e-07)	15.88%	+ NONE	15.88%
2	D.rs776746 (2.3e-05) + R.rs776746 (2.5e-07)	13.03%	+ R.rs7853758 (0.047) + R. rs914189 (0.0003)	17.92%
3	D.rs776746 (6.5e-07) +R.rs776746 (6.3e-10)	18.13%	+ R.rs7853758 (0.0391) + R. rs914189 (0.0019)	21.76%
4	D.rs776746 (1.6e-07) + R.rs776746 (1.2e-07)	17.74%	+ R.rs7853758 (0.0216)	19.2%

Likewise, for patients under paired-EIP classification, the regression model results showed that minor effect loci can further divide subgroups (Table 4). In the EM group, rs3775770 on SULT1E1 showed a significant association (P = 0.0092, 0.0166, and 0.0524) and accounted for 9.14%, 7.56% and 5.26% in the second, third, and fourth weeks post operation, respectively. Simultaneously, the minor effect locus rs7141505 on SLC7A8 not only showed a high association with CDRs (P = 0.0293, 0.0014, and 4.8e-07) in the paired-PM group, but the integrated model with other loci also accounted for a higher percentage of variance (3.15%, 14.07% and 36.03%) in the second, third, and fourth weeks, respectively.

**TABLE 4:** Linear regression model in paired-EM & paired-PM patients.

EIP Group	Week	Significant SNPs with P-value	Adjusted R^2^
Paired-EM Group	1	NONE	NONE
	2	SULT1E1 R.rs3775770 (0.0092)	9.14%
	3	SULT1E1 R.rs3775770 (0.0166)	7.56%
	4	SULT1E1 R.rs3775770 (0.0524)	5.26%
Paired-PM Group	1	NONE	NONE
	2	SLC7A8 D.rs7141505 (0.0293)	3.15%
	3	SLC7A8 D.rs7141505 (0.0014)	14.07%
	4	SLC7A8 D.rs7141505 (4.8e-07)	36.03%

### 3.5 High concordance between retrospective clinical-FIS classification and prospective genetic-EIP classification

**FIGURE 3:**
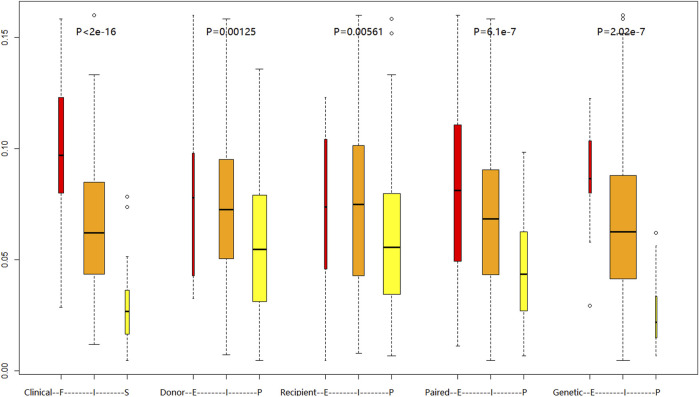
Comparison of daily tacrolimus dose requirements (mg/day) at postoperative week 4 across different metabolic classification systems:Clinical FIS phenotyping (Slow/Intermediate/Fast), Donor CYP3A5 rs776746 genotype, Recipient CYP3A5 rs776746 genotype, Combined donor-recipient CYP3A5 rs776746, Novel EIP genotyping. Boxplots show median (central line), IQR (box), and range (whiskers). Kruskal–Wallis test with Dunn’s correction was used for group comparisons.

After a set of polymorphisms that influenced tacrolimus elimination was screened and validated, a new EIP classification was built based on major effect genetic factors (donor and recipient CYP3A5 rs776746) and genetic factors with minor effects and fine regulation (recipient SULT1E1 rs3775770, donor SLC7A8 rs7141505), and can further subdivide groups by paired-EIP classification. Analysis of variance classification systems showed (Figure 3) that the immune balance dose in different patients groups by genetic-EIP classification have more obvious difference (P = 2.02e-7) than those by other classification, and is closer to value in clinical-FIS. This newly established genetic-EIP classification allows 73.2% overall consistency with the former retrospective clinical-FIS classification (Table 5). The agreement between genetic-EMs with clinical-FE patients is up to 79.17%, while the agreement between genetic-PMs with clinical-SE patients reached 61.11%. This finding demonstrates the important clinical value of our new prognostic classification based on multiple genes and takes into account the genetic diversity of donors and recipients.

**TABLE 5:** Agreement between retrospective clinical-FIS classification and each EIP classification.

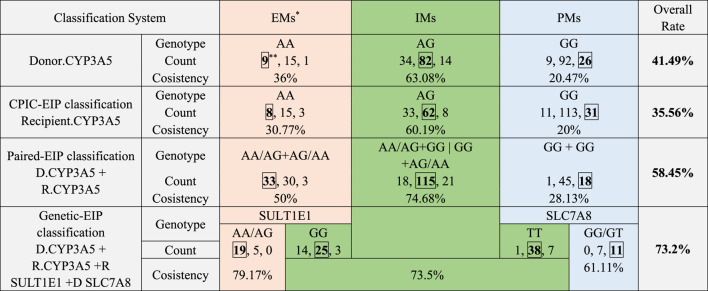

^a^ Patients classified into clinical-FM, clinical-IM, and clinical-SM, using each EIP, classification were colored respectively in red, green and blue.

^b^ Consistent patient number between clinical-FIS, and each EIP, classification was marked with box.

## 4 Discussion

Since precise dosing of the immunosuppressive drug tacrolimus after allogeneic liver transplantation is very important for reducing side effects and increasing the quality of long-term prognosis, the only clinical guideline for CYP3A5 genotype and tacrolimus dosing was released in 2015 by the Clinical Pharmacogenetics Implementation Consortium (CPIC) and aimed at kidney, heart, lung, and hematopoietic stem cell transplant patients and liver transplants in which the donor and recipient genotypes are identical. It is stated in the guideline that there are 3 classifications of dosing recommendations for tacrolimus based on CYP3A5 phenotype of transplant patients: Extensive metabolizer (EM) and Intermediate metabolizer (IM) were CYP3A5 expressers, who had lower tacrolimus trough blood concentrations and should be given an increased starting dose 1.5–2 times that of the recommended starting dose. Poor metabolizers (PM) were CYP3A5 non-expressers who had higher tacrolimus trough blood concentrations and should initiate therapy with the standard recommended dose. However, there are two major shortcomings in this CPIC guideline. One is that the donor and recipient genetic factors are not taken into consideration comprehensively, as the genotypes of donor liver and recipient intestine are different in most allogeneic liver transplantation cases, the former of which may also play an important roles as well as their combination. The other is that the CPIC guidelines are not specifically designed for liver transplantation. Considering the liver as an immune-preferred organ, liver transplantation is different from any other type of organ transplants. Moreover, patients in the early period after surgery always have liver insufficiency, and an excessive drug dose can easily increase liver damage and lead to poisoning. Reaching the targeted daily dose too early in the liver regeneration phase (convalescence phase, C. phase) easily led to a high trough blood concentration resulting from poor liver metabolic function. Given this information, we think that the current CPIC guidelines, especially the high initial recommended doses, are not suitable for most liver transplants and that small doses may be safer and more reliable for preventing postoperative complications (such as infection, liver dysfunction, renal insufficiency, diabetes, and tumor recurrence) in liver transplantation recipients (Lee et al., 2025; Lawendy et al., 2021; Mauro et al., 2024).

Furthermore, due to genetic factors, differences in drug dosage also exist between different ethnicities. From our clinical data, the tacrolimus dosage for our Chinese patients needed to maintain immune balance is generally half or even less than that of European and American patients. However, CYP3A5 and CYP3A4, as the main metabolic enzymes of tacrolimus in the liver and intestine, have similar mutation frequencies in Chinese and other populations (EAS_AF = 0.2867, AMR_AF = 0.2032, AFR_AF = 0.82, EUR_AF = 0.0567, SAS_AF = 0.3323), indicating that other genes may play an important role in tacrolimus metabolism. The minor allele frequencies of the loci on these unknown genes could vary widely among different populations, and the genotype of Caucasian patients may accelerate tacrolimus elimination and improve the rate overall, while the genotype of Chinese patients causes the opposite effect. Considering that the DMET array contains almost all important drug metabolism loci, we first looked at differences in the minor allele frequency on the DMET array among 5 populations in 1000G data (Choi et al., 2017; Gim et al., 2020; Michael et al., 2021). The result showed 26 loci with DIFF>0.6 among 5 populations, while only 5 loci had DIFF>0.5 between EAS and AMR populations, and all were related to alcohol metabolism, including rs3762894 on ADH4 (alcohol dehydrogenase 4), rs10008281 on ADH6 (alcohol dehydrogenase 6), rs1229984 on ADH1B (alcohol dehydrogenase 1B), rs886205 on ALDH2 (aldehyde dehydrogenase 2 family member), and rs11150606 on PRSS53 (serine protease 53). Protein-protein interaction network analysis did not indicate that these loci affect the CYP3A4 or CYP3A5 genes (See Supplementary Figure S6). When expanding to DIFF>0.4 between EAS and AMR populations, more loci and genes were found, including CYP2D6, which reflects both alcohol dehydrogenase and P450 families (see Supplementary Figure S7). These findings may explain the difference in tacrolimus elimination between our Chinese populations and Caucasian or other populations; however, a difference in the tacrolimus elimination rate also exists even within the Chinese population, further indicating that in addition to CYP3A5, comprehensive genetic loci research should be conducted to identify other minor metabolism-related genes and loci.

Moreover, it is more complicated to study tacrolimus metabolism for liver transplantation patients, as the genotypes of donors and their related recipients are always different. Recent studies have confirmed that both donor and recipient CYP3A5 play roles, but this finding does not perform well in clinical dosing regimens. Based on this consideration, a total of 284 pairs of donors and recipients were collected from three different centers to systematically assess the influence of genetic factors on tacrolimus elimination in the early postoperative period in Chinese liver transplant patients, and it is by far the largest data set to comprehensively analyze the genetic factors of tacrolimus medication in the Chinese population. Clinical information including drug dosage, trough blood concentrations, alanine aminotransferase and total bilirubin at 28 days postoperatively were analyzed and presented a complete picture of the Chinese population. CDRs are a link between genotype and the recommended dose and can better reflect the drug elimination rate. In the present study, we first established a clinical fast-intermediate-slow elimination classification (clinical-FIS classification) based on CDRs under TDM and explored its pharmacological and clinical significance. The stable doses of tacrolimus were 0.101 ± 0.036 mg/kg, 0.068 ± 0.039 mg/kg, and 0.038 ± 0.029 mg/kg in the clinical-FMs, clinical-IMs and clinical-SMs, respectively. The doses of the clinical-FMs, clinical-IMs and clinical-SMs continued to increase within the first 2 weeks post transplantation and then achieved target tacrolimus trough blood concentrations. The trend of the change in tacrolimus daily dose could be due to the recovery process of liver function in the early period after liver transplantation. The clinical-SMs had higher drug concentrations, alanine aminotransferase and creatinine levels than the clinical-FE and clinical-IE groups. This finding suggested that the TDM strategy had noticeable defects in the early period post liver transplantation. The clinical-SMs under TDM had delayed recovery of liver and kidney function and higher infection rate due to high tacrolimus trough blood concentration in early postoperative period. We consider this clinical-FIS classification was well described the clinical status and the clinical-SE group had bad clinical effect and should been predicted using pharmacogenomics strategy. Moreover, an increasing number of studies have found that early liver function after liver transplantation plays an important role in future prognosis and toxic side effects. Therefore, accurately administering the drug in the short period of time after surgery and achieving immune balance as soon as possible become the top priority of postoperative medication.

However, as allogeneic liver transplantation includes two genomes from both the donor and recipient, it may be necessary to account for both the donor and recipient genotypes when determining the dose (Liu et al., 2019; Wu et al., 2021; Coller et al., 2019). Studies to date have been inconclusive regarding the relative influence of the donor and recipient genotypes and whether donor liver and recipient intestinal genotypes come into play at different points post transplantation. Although some studies show that the donor genotype affects CDR from the first week post transplantation, others show that it does not begin to play a role until the second week or even the sixth month post transplantation (Huang et al., 2021; Monostory et al., 2015). Evidence is also conflicting for the recipient intestinal genotype: a few studies show that it never significantly affects tacrolimus concentrations, whereas others show that its influence on concentration is substantial only up to the point at which the donor genotype becomes important (Niu et al., 2023; Li et al., 2023). To fully understand the drug metabolism-related genetic loci of both donors and recipients, an association analysis between genotypes and CDR was conducted in liver transplantation recipients from 3 centers. Donors and recipient liver samples in Group 1 were first genotyped using the Affymetrix DMET Plus microarray, and CYP3A5 was found to have the most significant correlation with tacrolimus elimination both in donor and recipient genomes. In addition to rs776746 on CYP3A5, the CYP3A4*1G allele (rs2242480), a novel G-to-A substitution at position 82,266 in intron 10, has been identified to have the highest contribution of total variance in all patient samples. Several studies have already indicated that this SNP can increase the activity of the CYP3A4 enzyme and is related to the pharmacokinetics of tacrolimus, in addition to being responsible for the interindividual differences in cyclosporine disposition (Zhou et al., 2025; Zhai et al., 2024). However, most of the predictive value of recipient CYP3A4 rs2242480 on tacrolimus elimination overlapped with recipient CYP3A5 rs776746, as these two loci are in linkage disequilibrium (R-square = 0.52). This observation may be because linkage disequilibrium exists between CYP3A5 and CYP3A4, the latter of which is the main metabolic protein of tacrolimus in the kidney. Therefore, a paired-EIP classification was constructed based on both the donor and recipient CYP3A5 rs776746 genotype and includes an extensive metabolizer (paired-EM, recipient of CYP3A5 rs776746 AA/AG with CYP3A5 rs776746 AA/AG donor), intermediate metabolizer (paired-IM, recipient of CYP3A5 rs776746GG with CYP3A5 rs776746 AA/AG donor or recipient of CYP3A5 rs776746 AA/AG with CYP3A5 rs776746GG donor), and poor metabolizer (paired-PM, recipient of CYP3A5 rs776746GG with CYP3A5 rs776746GG donor). We compared the grouping results of this paired-EIP classification with the previously established clinical-FIS classification and found that their agreement was only 58.45%, although it was higher than those with single donor CYP3a5 genotype (41.49%) or single recipient genotype (35.56%) alone. However, from a practical point of view, the results of paired-EIP classification was far from satisfied. In more detail, the agreement between clinical-FM and paired-EM and the agreement between clinical-SM and paired-PM is too low for dosage guidance in clinical applications. Moreover, paired-PMs even showed diverse tacrolimus metabolic rates (see Supplementary Figure S4), prompting us to consider looking for minor effect gene loci in the absence of a strong CYP3A5 influence.

Association analysis was then performed independently in paired-EMs, paired-IMs and paired-PMs to find minor effect loci for subdividing separate groups. The initially screened sites were further validated in the patients from Centers 2 and 3, and ultimately, 10 loci were shown to have significant correlation in Table 2. Though loci on genes SLC28 and ABCG1 had low P values, the linear regression analysis results showed that these loci provided a minor contribution to CDR variance (see Table 3). By contrast, in the patient subgroups, where the influence of CYP3A5 was eliminated between populations, the impacts of minor effect loci gradually appear instead. SULT1E1 was the most significant locus in paired-EM patients and had a strong contribution to the variance (see Table 4). Estrogen sulfate transferase SULT1E1 is the key enzyme of the sulfation reaction, which is the main pathway in estrogen metabolism. Previous *in vitro* studies demonstrate bidirectional tacrolimus-estrogen interactions: *In vitro* studies utilizing human and animal hepatic/intestinal microsomes have demonstrated that ethinylestradiol significantly inhibits tacrolimus metabolism through competitive inhibition of CYP3A4. Conversely, experiments with human and recombinant hepatic microsomes revealed that tacrolimus itself concentration-dependently suppresses phase I estradiol metabolism, particularly 2-hydroxylation. It is speculated that SULT1E1 changes the metabolism level of estrogen and thus affects tacrolimus metabolism (Niu et al., 2025; Ghadimi et al., 2018). Remarkably, SLC7A8 was also validated to subdivide paired-PM patients. This protein is an amino acid transporter that increases the reabsorption of levodopa in the kidneys and is a key step in the synthesis of dopamine in the kidneys (Tina et al., 2018). Although there are no direct studies on SLC7A8’s involvement in tacrolimus metabolism, emerging evidence has gradually elucidated the impact of drug transporter genes and their genetic polymorphisms on tacrolimus pharmacokinetics (Liu et al., 2017). As shown in our data, donor and recipient CYP3A5 rs776746 were major genetic factors influencing tacrolimus elimination, and recipient SULT1E1 rs3775770 and donor SLC7A8 rs7141505 were minor genetic factors influencing tacrolimus elimination. A novel genetic-EIP classification based on multiple genetic loci, including donor and recipient CYP3A5 rs776746, recipient SULT1E1 rs3775770, and donor SLC7A8 rs7141505, was constructed to predict tacrolimus elimination. This genetic-EIP classification shows 73.2% overall agreement with former retrospective clinical-FIS classification (Table 5). Furthermore, the agreement between genetic-EMs and the clinical-FMs and the agreement between genetic-PMs and the clinical-SMs can be as high as 79.17% and 61.11%, respectively, which could provide an effective reference for clinical trials.

This approach holds particular clinical significance for predicting side effects (SEs), as it enables more precise dose individualization to minimize toxicity risks while maintaining therapeutic efficacy.When genetic testing indicates a high risk of SE, we recommend the following personalized dosing strategies: 1) Reducing the initial postoperative dose; 2) Exercising greater caution in subsequent dose adjustments; 3) Enhancing therapeutic drug monitoring (TDM) to avoid excessive concentrations. This pharmacogenomics-guided, preemptive dosing strategy offers a novel approach to precision medicine in tacrolimus administration after liver transplantation.

Characteristics of experimental design and statistical methods in this study: Our study differs from traditional genetic association analyses in the following aspects, which enhance the robustness and clinical relevance of our findings: Two-Step SNP Screening: To minimize false-positive associations, we employed a training-validation cohort design. This approach reduces Type I error rates compared to single-cohort analyses. Unlike cross-sectional studies, we collected tacrolimus concentrations at multiple time points (Days 7, 14, 21, and 28 post-transplantation) to capture dynamic dose-concentration relationships. The multicenter design, with a large sample size (n = 284) and log2-transformation of drug concentrations, effectively reduced inter-center systematic biases arising from differences in assay protocols or calibration standards. These methodological refinements improve the generalizability of our conclusions to real-world clinical settings.

To our knowledge, this study was the first in-depth and comprehensive assessment of the influence of several EIP classifications, including donor and recipient CYP3A5 rs776746 polymorphisms, on tacrolimus clearance in the early postoperative period in Chinese liver transplantation patients. In the 3 independent cohorts of recipients from different transplant centers, the paired-EIP classification has already shown a stronger distinguishing ability than CPIC-EIP with only donor CYP3A5 rs776746 or recipient CYP3A5 rs776746 genotype and is more precise and effective in guiding individualized tacrolimus use. In addition to the well-known major CYP3A5 rs776746 site, other minor alleles, such as recipient SULT1E1 rs3775770 and donor SLC7A8 rs7141505, were provided here as novel potential genetic biomarkers of tacrolimus elimination and biological mechanisms. Thus, considering those loci, the novel genetic-EIP classification could be a good supplement to CPIC guidelines to create individualized treatment plans and make the medication guidance system more precise. In particular, the clinical dose of tacrolimus in the Chinese population is half or even less than that of the Caucasian population.

Therefore, clinical drug delivery in the Chinese population requires more precise and finer regulation than Caucasians. The impact of the new genetic polymorphisms found in our study on the early postoperative tacrolimus clearance in Chinese liver transplant patients undoubtedly compensates for the inadequacy of the old CPIC guidelines. A simple-to-use panel including these contributing loci will be designed for further clinical trials. This layered analysis from the external clinical phenotype to the intrinsic molecular genetic information is not only a supplement to and subdivision of traditional clinical grouping by molecular biology typing but also an effective attempt to summarize forward-looking research methods from the experience of retrospective studies.

## Data Availability

The original contributions presented in the study are publicly available. This data can be found here: https://www.ncbi.nlm.nih.gov/geo/, accession number GSE53792.
