# Supplementary material for: Clinical significance and gene prediction of a novel classification system based on tacrolimus concentration-to-dose ratio in the early post-liver transplant period
Source: Front Pharmacol. 2025 Jul 21;16:1614753. doi: 10.3389/fphar.2025.1614753 (PMC12319242; doi:10.3389/fphar.2025.1614753)

Supplementary Table1 Varied distribution of DMET loci in 6 populations from 1000G

| Diff=MAX(MAF)-MIN(MAF) | SNP |
| --- | --- |
| <0.1 | 127 |
| [0.1, 0.2) | 151 |
| [0.2, 0.3) | 163 |
| [0.3, 0.4) | 114 |
| [0.4, 0.5) | 82 |
| >=0.5 | 96 |
| >=0.8 | 6 |

Supplementary Figure1 Change characteristics of pharmacological parameters and liver function in the early postoperative period in liver transplantation patients

C

B

A

D

E

Supplementary Figure2 Logarithm and Normalization of C/D ratio


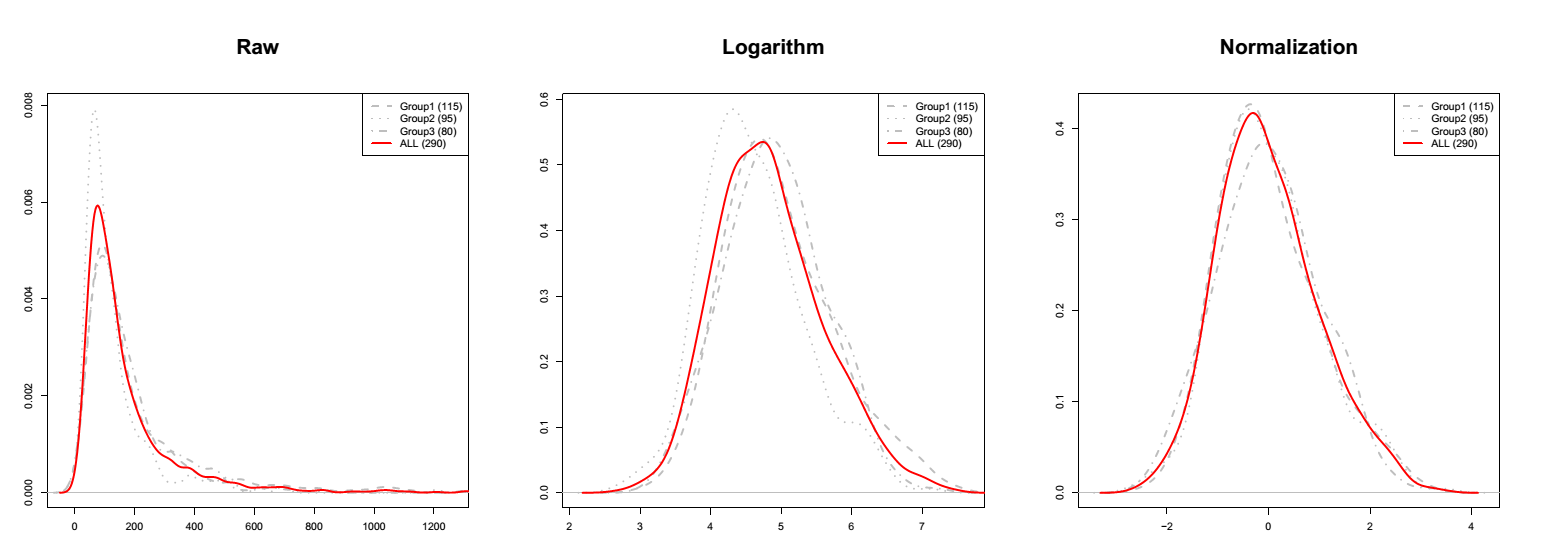


Supplementary Figure3 Boxplot of CDRs in Clinical-FIS Groups


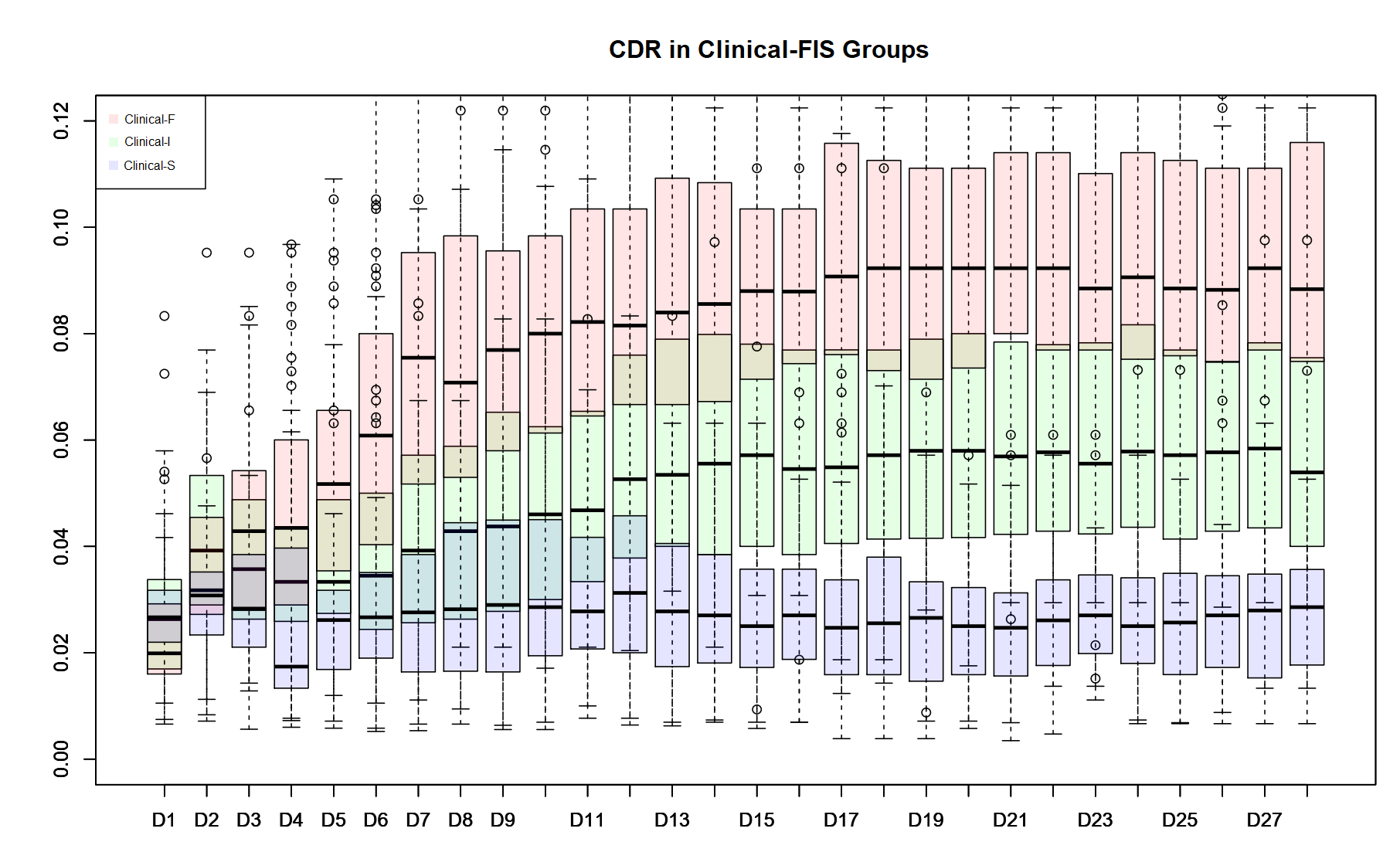


Supplementary Figure4 Boxplot of CDR in patients with different donor and recipient CYP3A5 genotype


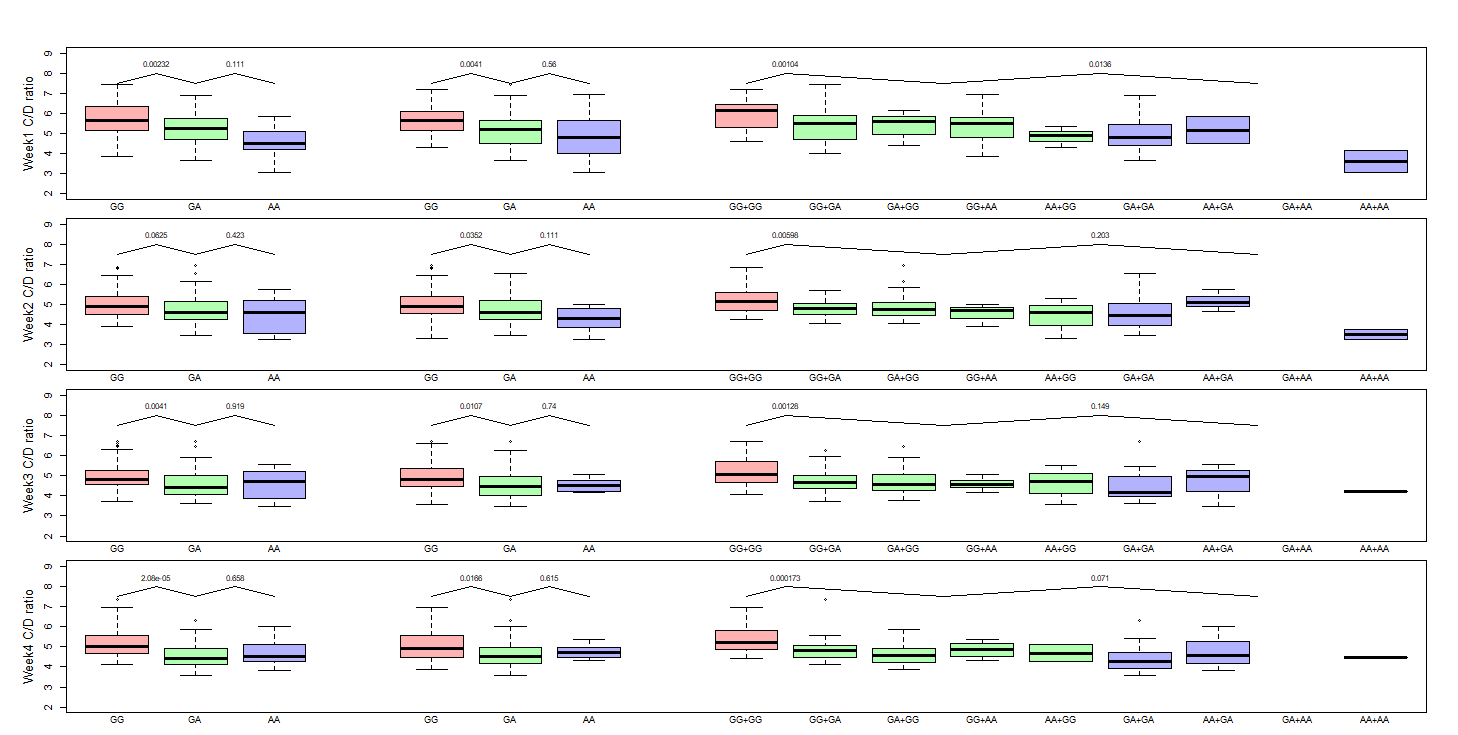


Supplementary Figure5 Boxplot of CDR in different patient groups by CPIC-EIP, paired-EIP and genetic-EIP respectively.


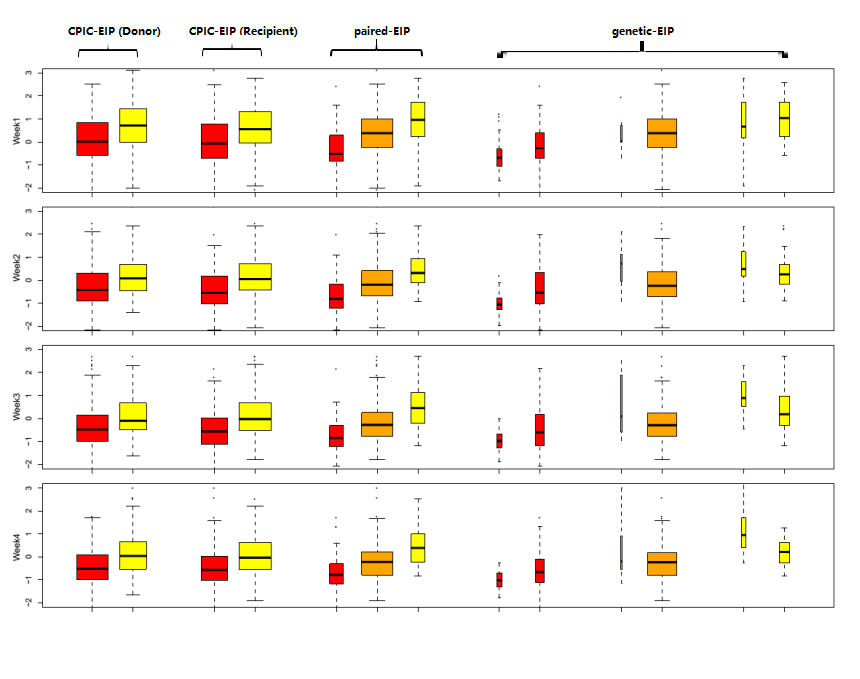


Supplementary Figure6 Protein-protein interaction network of DMET genes with DIFF>0.6.


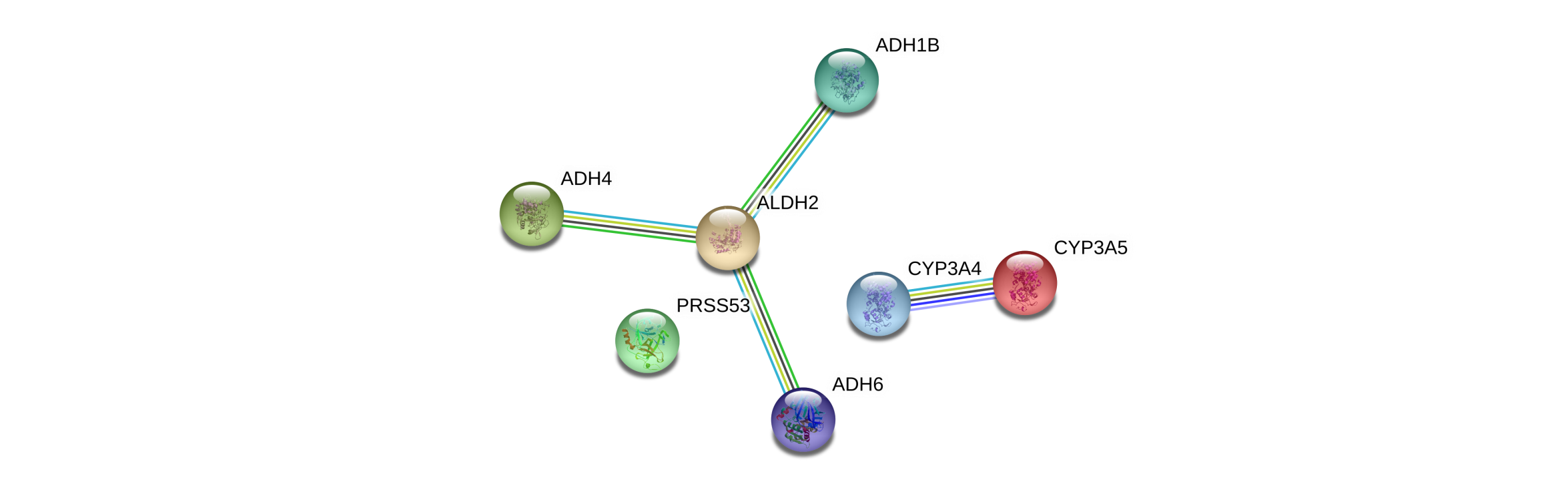


Supplementary Figure 7 Protein-protein interaction network of DMET genes with DIFF>0.4.


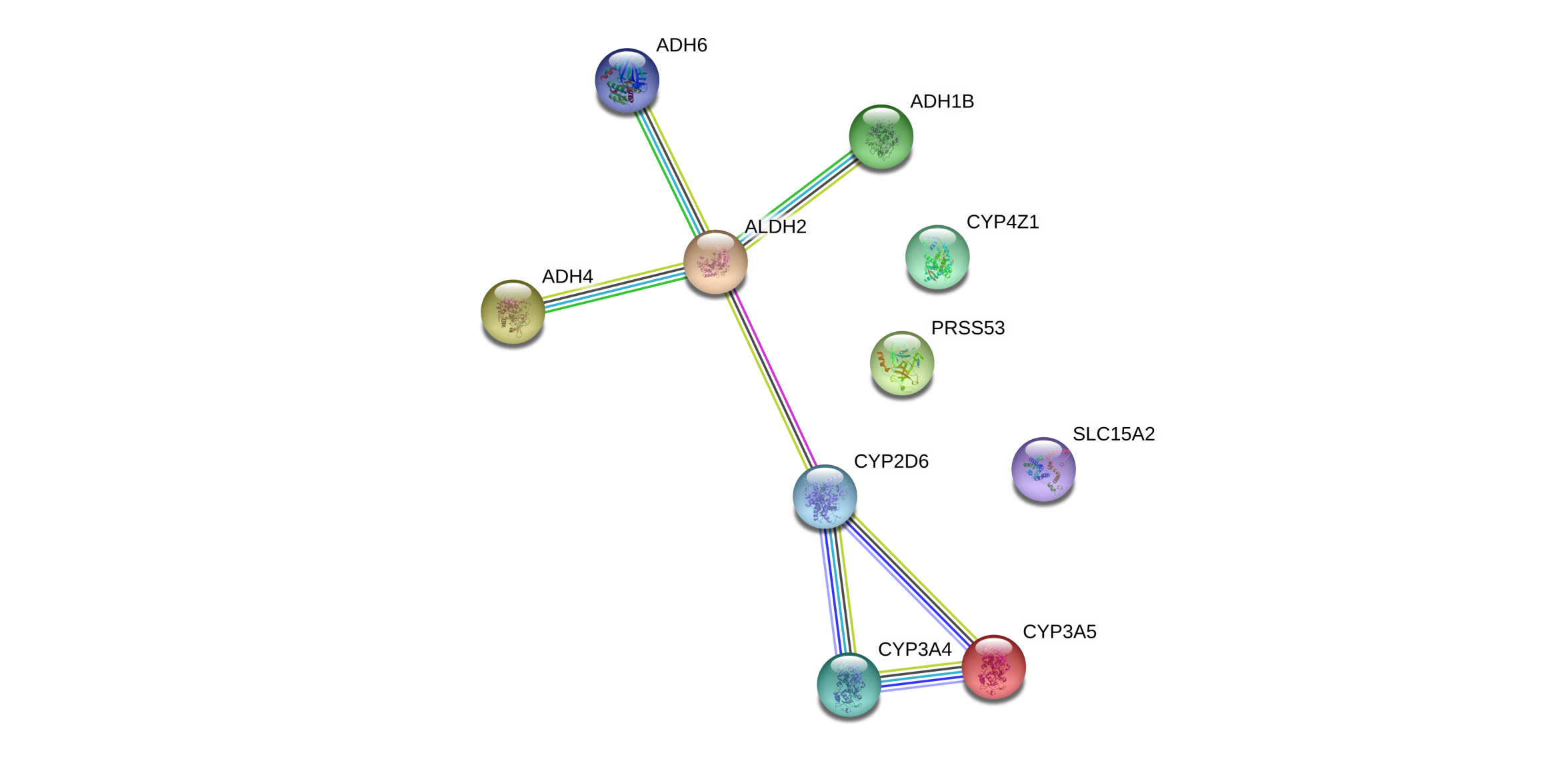

Supplement: Supplementary file 3 [file DataSheet1.docx]
